# The lncRNA H19 Promotes Cell Proliferation by Competitively Binding to miR-200a and Derepressing *β*-Catenin Expression in Colorectal Cancer

**DOI:** 10.1155/2017/2767484

**Published:** 2017-01-10

**Authors:** Weiwei Yang, Ning Ning, Xiaoming Jin

**Affiliations:** ^1^Department of Pathology, Harbin Medical University, Harbin 150081, China; ^2^Department of Gastrointestinal Surgery, Peking University International Hospital, Beijing 102206, China

## Abstract

H19, a paternally imprinted noncoding RNA, has been found to be overexpressed in various cancers, including colorectal cancer (CRC), and may function as an oncogene. However, the mechanism by which H19 regulates CRC progression remains poorly understood. In this study, we aimed to assess H19 expression levels in CRC tissues, determine the effect of H19 on CRC proliferation, and explore the mechanism by which H19 regulates the proliferation of CRC. We measured H19 expression using qRT-PCR and analysed the effects of H19 on colon cancer cell proliferation via cell growth curve, cell viability assay, and colony formation assays. To elucidate the mechanism underlying these effects, we analysed the interactions between H19 and miRNAs and identified the target gene to which H19 and miRNA competitively bind using a series of molecular biological techniques. H19 expression was upregulated in CRC tissues compared with adjacent noncancerous tissues. H19 overexpression facilitated colon cancer cell proliferation, whereas H19 knockdown inhibited cell proliferation. miR-200a bound to H19 and inhibited its expression, thereby decreasing CRC cell proliferation. *β*-Catenin was identified as a target gene of miR-200a. H19 regulated *β*-catenin expression and activity by competitively binding to miR-200a. H19 promotes cell proliferation by competitively binding to miR-200a and derepressing *β*-catenin in CRC.

## 1. Introduction

Colorectal cancer (CRC) is the third-most common cancer and the fourth-most common cause of cancer-related death worldwide [1]. A significant proportion of CRC patients experience recurrence and die within 5 years after surgical treatment of their primary tumour [2]. Thus, it is important to elucidate the molecular mechanisms underlying CRC proliferation.

Long noncoding RNAs (lncRNAs, >200 nucleotides in length) have limited or no protein-coding capacity [3, 4]. Previous studies have demonstrated that lncRNAs are crucial molecules in human malignancies [5–7]. Several studies have revealed that lncRNAs can regulate gene expression at the transcriptional, posttranscriptional, and epigenetic levels [8, 9]. For example, the lncRNA SPRY4-IT1 can promote CRC metastasis by regulating the epithelial-mesenchymal transition [10]. Furthermore, lncRNAs are required for CRC cell proliferation and migration [11], are associated with a poor prognosis of CRC [12], and promote colon tumourigenesis [13].

The H19 gene is a conserved and maternally expressed gene located on human chromosome 11p15 that plays key roles in embryonal development and growth control [14, 15]. The results of functional studies suggest that H19 acts as an oncogene to initiate and promote the progression of malignancies, such as breast cancer [16], oesophageal cancer [17], bladder cancer [18], and CRC [19]. However, the mechanisms by which H19 promotes cancer progression are not well understood; in particular, the molecular mechanisms by which H19 regulates CRC proliferation are unknown. In this study, we determined that H19 competitively binds to miR-200a, suggesting that H19-mediated derepression of *β*-catenin is a potential mechanism by which this lncRNA promotes cell proliferation in CRC.

## 2. Materials and Methods

### 2.1. CRC Tissue Samples

Thirty CRC tissue samples and matched adjacent normal tissue samples were obtained from patients who underwent surgery at the Affiliated Tumor Hospital of Harbin Medical University. All the tissues were diagnosed by two independent pathologists. Each patient provided written informed consent to participate in this study, which was approved by the Ethics Committee of Harbin Medical University. All the tissue samples were immediately frozen in liquid nitrogen and stored at −80°C until RNA extraction.

### 2.2. Cell Culture

The human colorectal cancer cell lines HCT116 and SW480 and human embryonic kidney 293TN (HEK-293TN) were purchased from the Institute of Biochemistry and Cell Biology, Shanghai Institutes for Biological Sciences, CAS. The HCT116 and SW480 cells were cultured in RPMI 1640 medium (Gibco, USA), and the HEK-293TN cells were maintained in DMEM (Gibco) supplemented with 10% FBS (Gibco) and 2% penicillin-streptomycin (10 U/mL) at 37°C in a 5% CO_2_ atmosphere.

### 2.3. Cell Growth Curve

After 48 h of transfection, human CRC cells were seeded into 6-well plates at a density of 1 × 10^5^ cells/2 mL of complete medium per well and incubated at 37°C in 5% CO_2_. The cells were counted every 24 hours. All experiments were performed in triplicate and repeated three times.

### 2.4. Cell Viability Assay

The cells were seeded in 96-well plate. Cellular viability was measured using luciferase-based ATP quantitation assay (CellTiter-Glo™, Promega, USA) following the manual at 6 h (used to reflect the initial cell number, shown as 0 h in the result), 24 h, 48 h, and 72 h. Briefly, equilibrate the plate at RT for 30 min, add 100 *μ*L CellTiter-Glo Reagent in each well, and mix contents on an orbital shaker. Incubate the plate at RT for 10 min and record luminescence under spectra Max M5 microplate reader (Molecular Devices, USA). The value was used to reflect the relative cell viability. All experiments were performed in triplicate and repeated three times.

### 2.5. Plate Colony Formation Assay

Briefly, after 48 h of transfection, 1 × 10^3^ human CRC cells were initially seeded into each well of a six-well plate and maintained in medium containing 10% FBS, which was refreshed every two days. After the cells had incubated for 14 days at 37°C in 5% CO_2_, their colonies were visible to the naked eye. The cells were fixed with methanol and stained with 1% crystal violet for 15 min before being counted. The colony numbers were counted using ImageJ software. All experiments were repeated three times.

### 2.6. Soft-Agar Colony Formation Assay

1 mL of 0.6% agarose gel with 1 × RPMI 1640 complete medium was placed into 6-well plates. Then cells (1 × 10^3^ per well) were mixed with 0.3% agarose in growth medium, plated on top of a solidified layer. These cells were continued to be cultured for 14 days at 37°C in a humidified atmosphere containing 5% CO_2_. The colony numbers were counted using the same method as above. All experiments were repeated three times.

### 2.7. Western Blot Analysis

Total protein was extracted from the cells using RIPA buffer (Beyotime, China). Equal amounts of protein were loaded into each lane, resolved via SDS-PAGE, and electrophoretically transferred to PVDF membranes (Bio-Rad), which were subsequently incubated in 5% nonfat milk dissolved in Tris-buffered saline (TBS) containing 0.1% Tween-20 for 1 h at room temperature, followed by incubation with anti-*β*-catenin (1 : 1000, Abcam, USA) or anti-actin antibodies (1 : 2000, Abcam, USA) overnight at 4°C. Then, the membranes were incubated with peroxidase-conjugated goat anti-rabbit or anti-mouse secondary antibodies for 1 h after being washed. Immunoreactive bands were detected via enhanced chemiluminescence (ECL Kit, Beyotime, China).

### 2.8. Transfection

pWPXL lentiviral vectors were employed to overexpress H19 in HCT116 and SW480 cells, as previously described [20]. Briefly, the full-length sequence H19 was amplified via reverse-transcription PCR, digested, and inserted into the pWPXL vector. To package H19-overexpressing lentiviral particles, the pWPXL plasmid carrying full-length H19 was transfected into HEK-293TN cells together with packaging plasmids (pSPAX2 and pMD.2G) for 48 h. Then, the supernatant containing the pseudovirus was harvested and condensed using PEG. A small interfering RNA (siRNA) against H19 (siRNA sequence: GCAGGACAUGACAUGGUCC) and a nontargeted sequence (negative control, NC: UCCGCUGACGACAAGGAUG) were synthesized by GenePharma (Hangzhou, China), and the miR-200a mimic and miR-200a inhibitor were purchased from Invitrogen (Invitrogen, USA). These miRNAs and siRNAs were transfected into cells using Lipofectamine 2000 (Invitrogen, USA) according to the manufacturer's instructions. The primers used for lentiviral vector construction are presented in Table 1.

### 2.9. Quantitative Real-Time PCR (qRT-PCR)

Total RNA was extracted from CRC tissue samples and cultured cells using Trizol reagent (TaKaRa, Dalian, China), according to the manufacturer's instructions. Then, 2 *μ*g of total RNA was reverse transcribed into cDNA using a PrimeScript RT Master Mix Perfect Real Time Kit (TaKaRa). qRT-PCR was performed using ABI 7900 RT-PCR system with a SYBR Premix Ex Taq Kit (TaKaRa). The H19 and miR-200a primers were synthesized by GenePharma (Shanghai, China). The RT-PCR primers are presented in Table 1.

### 2.10. Luciferase Reporter Assay

Dual-reporter expression clones of the wild-type (WT) and mutant (MUT) *β*-catenin 3′-UTR were inserted into a pmirGlo Dual-Luciferase miRNA Target Expression Vector (Promega, USA). The sequences of these clones are presented in Table 1. These constructs allow firefly luciferase to serve as the miR 3′-UTR target reporter. The *β*-catenin 3′-UTR reporter plasmid and miR-200a were cotransfected into HEK-293TN cells, and luciferase activity was measured using a Dual-Luciferase Reporter Assay Kit (Promega, USA). TOP/FOP-Flash assay was also performed to examine *β*-catenin activity. The *β*-catenin reporter plasmid (TOP-Flash) and its mutant control (FOP-Flash) were purchased from Millipore Corporation. HCT116 cells were seeded into 24-well plates and maintained in complete RPMI 1640 medium. After reaching 60% confluence, the cells were transfected with TOP-Flash/FOP-Flash plasmids containing H19. After 24 h of cotransfection, luciferase activity was measured using a Dual-Luciferase Reporter Assay Kit. All experiments were performed in triplicate and repeated three times.

### 2.11. Statistical Analysis

All statistical analyses were performed using SPSS 17 software (SPSS, USA). The significance of the differences between groups was estimated using Student's *t*-test, and all results are reported as the mean ± SD. *P* < 0.05 or *P* < 0.01 was considered statistically significant.

## 3. Results

### 3.1. H19 Expression Is Significantly Elevated in CRC Tissues and Promotes CRC Cell Proliferation

**Figure 1:**
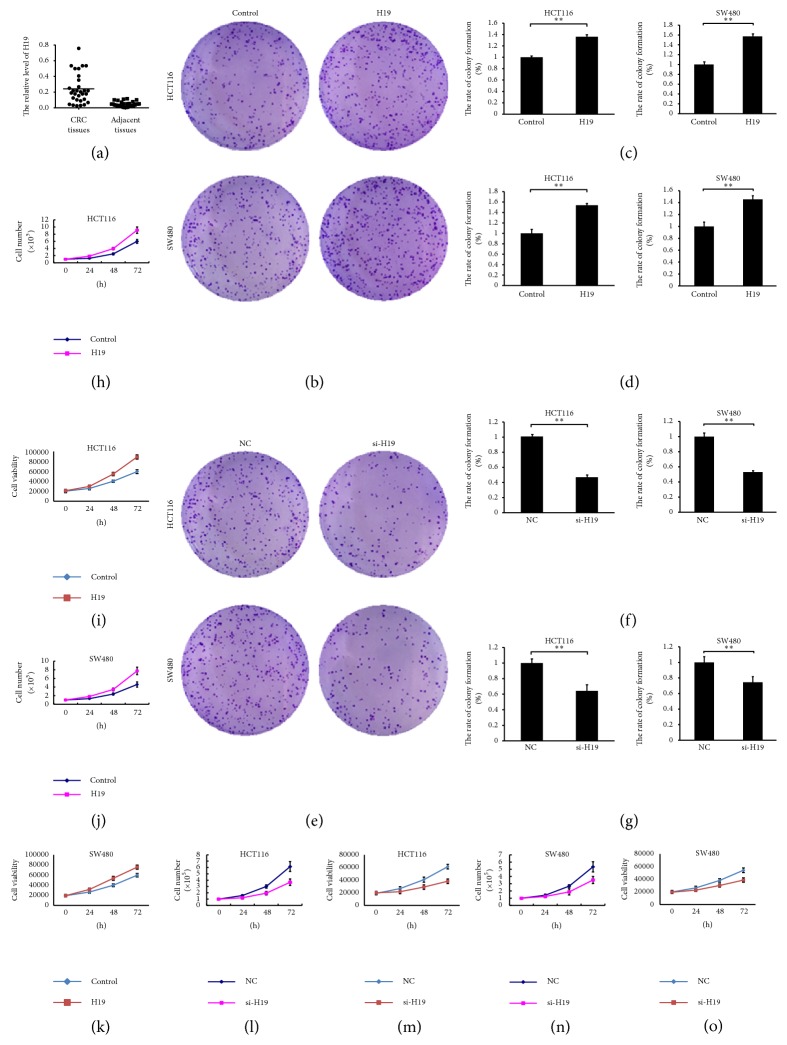
H19 is highly expressed in CRC tissues and promotes cell proliferation. (a) H19 expression is significantly increased in CRC tissues compared with adjacent normal tissues, as demonstrated by RT-PCR. ((b) and (c)) H19 overexpression increases the colony formation rate of HCT116 and SW480 cells (soft-agar assay, *P* < 0.01). (d) H19 overexpression increases the colony formation rate of HCT116 and SW480 cells (plate colony formation assays, *P* < 0.01). ((e) and (f)) H19 knockdown decreases the colony formation rate of HCT116 and SW480 cells (soft-agar assay, *P* < 0.01). (g) H19 knockdown decreases the colony formation rate of HCT116 and SW480 cells (plate colony formation assays, *P* < 0.01). ((h) and (i)) H19 overexpression promotes HCT116 cell proliferation (cell growth curve and cell viability assay, *P* < 0.05). ((j) and (k)) H19 overexpression promotes SW480 cell proliferation (cell growth curve and cell viability assay, *P* < 0.05). ((l) and (m)) H19 knockdown inhibits HCT116 cell proliferation (cell growth curve and cell viability assay, *P* < 0.05). ((n) and (o)) H19 knockdown inhibits SW480 cell proliferation (cell growth curve and cell viability assay, *P* < 0.05). “*∗∗*” refers to *P* < 0.01.

First, we measured the changes in H19 expression in CRC. Specifically, we employed qRT-PCR to detect H19 expression in 30 paired CRC and adjacent non-CRC tissue samples. Compared with adjacent non-CRC tissues, CRC tissues exhibited significantly elevated H19 expression levels (Figure 1(a), *P* < 0.01). To elucidate the role of H19 in CRC cell proliferation, we employed lentiviral vectors to overexpress H19 in HCT116 and SW480 cells. Our colony formation assays, cell growth curve, and cell viability assay demonstrated that forced H19 expression in the two cell lines promoted cell proliferation in both cell lines compared with the control treatment (Figures 1(b), 1(c), 1(d), 1(h), 1(i), 1(j), and 1(k), *P* < 0.05), whereas H19 knockdown significantly suppressed CRC cell proliferation in both cell lines compared with the control treatment (Figures 1(e), 1(f), 1(g), 1(l), 1(m), 1(n), and 1(o), *P* < 0.05). The colony formation assay was also performed with a soft-agar colony formation assay, and similar results to plate colony formation were obtained.

### 3.2. H19 Binds to miR-200a in CRC Cells, and miR-200a Inhibits H19 Expression and CRC Cell Proliferation

**Figure 2:**
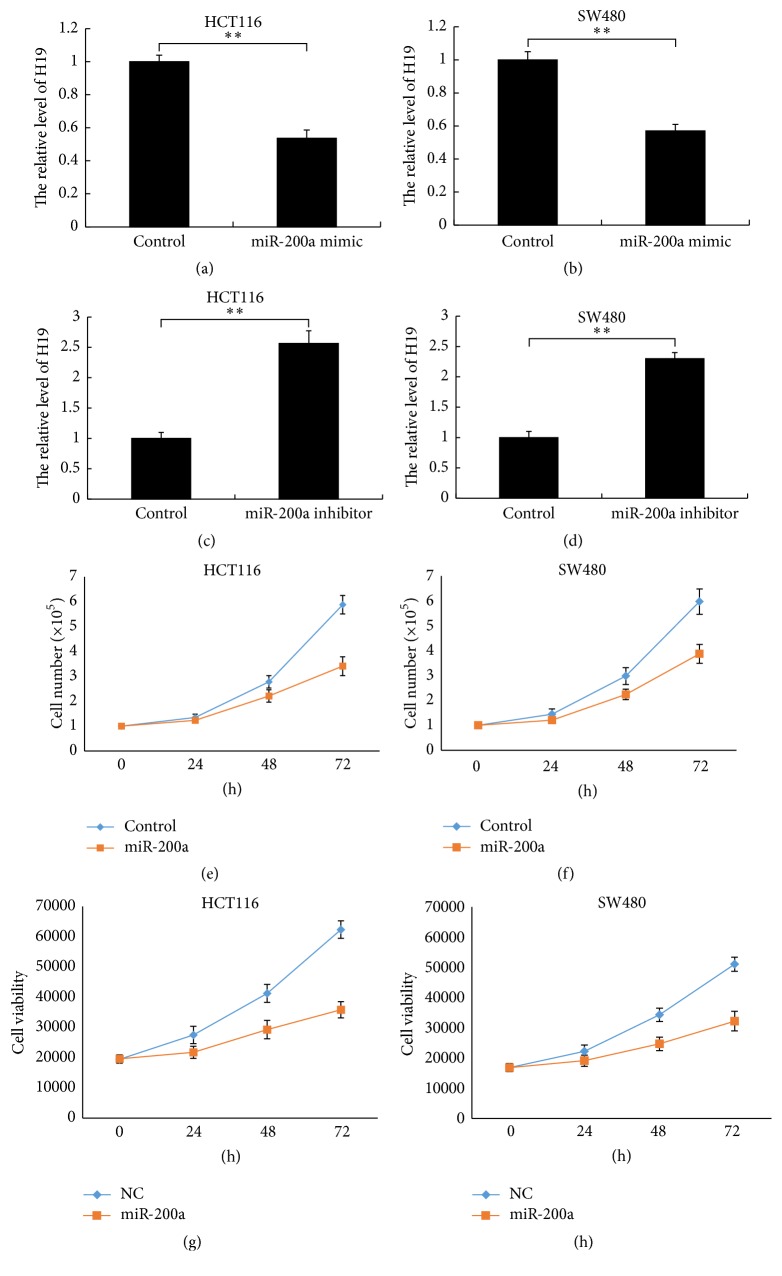
miR-200a regulates H19 expression in CRC cells. (a) Application of a miR-200a mimic decreases H19 levels in HCT116 cells (*P* < 0.01). (b) Application of a miR-200a mimic decreases H19 levels in SW480 cells (*P* < 0.01). (c) Application of a miR-200a inhibitor increases H19 levels in HCT116 cells (*P* < 0.01). (d) Application of a miR-200a inhibitor increases H19 levels in SW480 cells (*P* < 0.01). ((e) and (f)) miR-200a decreases HCT116 and SW480 cell proliferation (cell growth curve, *P* < 0.05). ((g) and (h)) miR-200a decreases HCT116 and SW480 cell proliferation (cell viability assay, *P* < 0.05). “*∗∗*” refers to *P* < 0.01.

To elucidate the mechanism by which H19 promotes cell proliferation and identify the possible downstream target of H19, we screened the potential miRNAs that bind to H19 using LncBase Predicted v.2 of DIANA tools. We discovered that miR-200a might interact with H19. To evaluate the relationship between H19 and miR-200a, we transfected a miR-200a mimic and miR-200a inhibitor into HCT116 and SW480 cells and analysed H19 expression in both cell lines via qRT-PCR after 48 h of transfection. The miR-200a mimic significantly decreased H19 expression levels in both cell lines compared with the control treatment (Figures 2(a) and 2(b), *P* < 0.01). Whereas miR-200a inhibitor increased H19 expression in both cell lines (Figures 2(c) and 2(d), *P* < 0.01). These results indicate that miR-200a reduces H19 expression after binding to H19 in CRC cells. Additionally, the results of our cell growth assay and cell viability assay indicate that miR-200a decreases CRC cell proliferation (Figures 2(e), 2(f), 2(g), and 2(h), *P* < 0.05).

### 3.3. miR-200a Represses *β*-Catenin Expression in CRC

**Figure 3:**
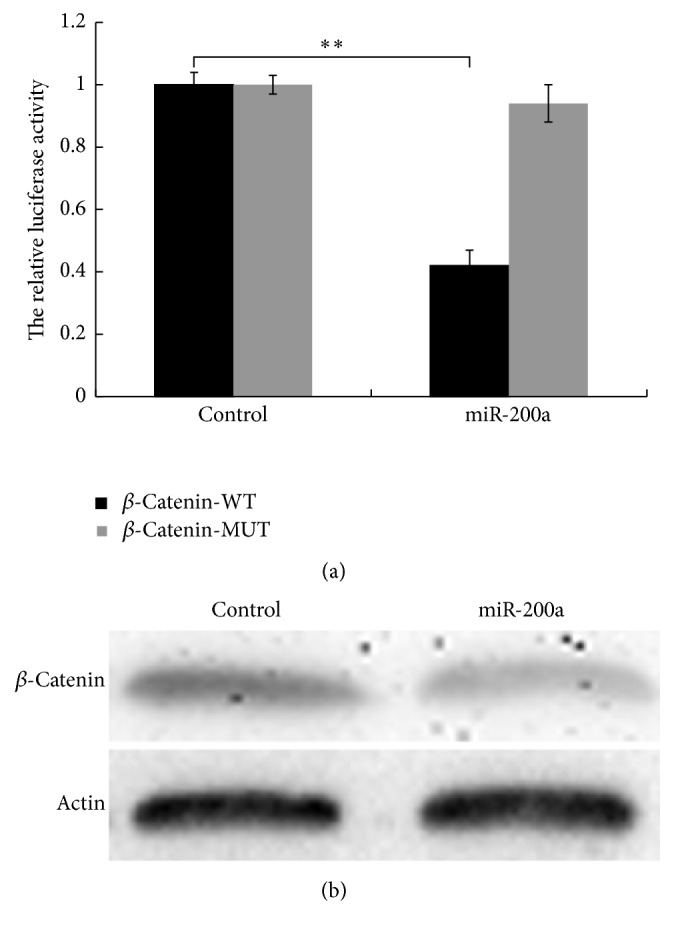
*β*-Catenin is a target of miR-200a, and miR-200a represses *β*-catenin expression in HCT116 cells. (a) miR-200a reduces the luciferase activity of a WT *β*-catenin reporter plasmid, as demonstrated via a luciferase reporter assay (*P* < 0.01). (b) miR-200a overexpression decreases *β*-catenin levels, as demonstrated via western blot analysis. “*∗∗*” refers to *P* < 0.01.

To identify the potential genes regulated by H19 via miR-200a, we used TarBase v7.0 of DIANA TOOLS to screen the potential targets of miR-200a and found that *β*-catenin was a candidate miR-200a target gene [21–23]. To conclusively determine whether *β*-catenin is a target of miR-200a, we performed a luciferase reporter assay and constructed luciferase reporter plasmids carrying the WT or MUT form of the *β*-catenin 3′-UTR. We measured luciferase activity at 24 h after cotransfection of luciferase reporter plasmids carrying miR-200a into HEK-293TN cells. The results demonstrated that miR-200a reduced the luciferase activity of only the WT *β*-catenin 3′-UTR (Figure 3(a), *P* < 0.01). We also evaluated *β*-catenin expression levels via western blot analysis, and the results confirmed that miR-200a overexpression significantly decreased *β*-catenin expression levels (Figure 3(b)).

### 3.4. H19 Elevates *β*-Catenin Expression and Activity in CRC Cells

**Figure 4:**
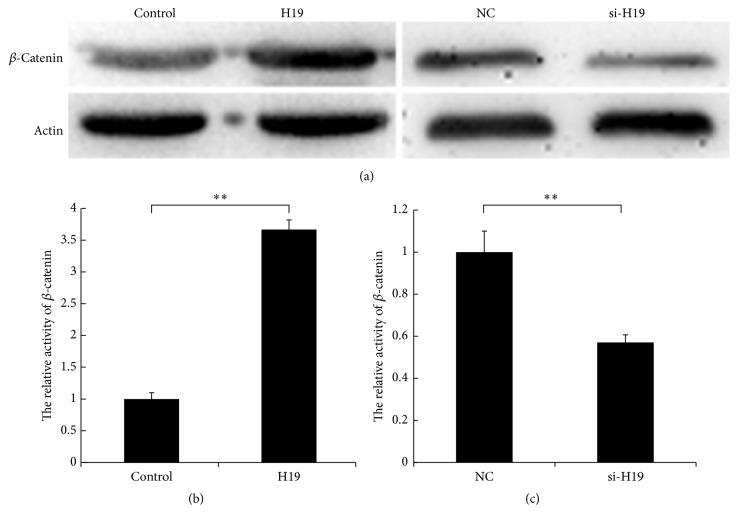
H19 increases *β*-catenin expression and activity in CRC cells. (a) H19 overexpression promotes *β*-catenin expression, whereas H19 knockdown decreases *β*-catenin expression. (b) H19 elevates *β*-catenin activity (*P* < 0.01). (c) H19 knockdown decreases *β*-catenin activity (*P* < 0.01). “*∗∗*” refers to *P* < 0.01.

To determine whether H19 promotes CRC proliferation through the miR-200a/*β*-catenin pathway, we examined the effects of H19 on *β*-catenin expression and activity in CRC. Specifically, we used western blot to detect *β*-catenin expression after H19 overexpression or knockdown in HCT116 and SW480 cells. As shown in Figure 4(a), H19 overexpression elevated *β*-catenin expression, whereas H19 knockdown decreased *β*-catenin expression in both cell lines. Furthermore, we transfected TOP-Flash or FOP-Flash plasmids into H19-overexpressing and H19-silenced HCT116 cells to evaluate the effect of H19 on the transactivation activity of *β*-catenin. Our results indicated that H19 increases TOP-Flash plasmid luciferase activity whereas H19 knockdown decreases TOP-Flash plasmid luciferase activity (Figures 4(b) and 4(c), *P* < 0.01). These results reveal that H19 competitively binds to miR-200a and derepresses *β*-catenin expression in CRC.

## 4. Discussion

The Wnt signalling pathway is responsible for initiating numerous triggers of CRC via active *β*-catenin [24]. Genome-wide data have recently revealed that the Wnt/*β*-catenin pathway is one of the pathways most frequently modified via genetic or epigenetic transformations in CRC, affecting nearly 90% of all Wnt/*β*-catenin gene family members [25]. Wnt signalling overactivation is a trait of CRC. The Wnt/*β*-catenin signalling pathway comprehensively participates in cell proliferation [26] and is involved in CRC metastasis [27]. Adenomatous polyposis coli (APC) is a tumour suppressor and a negative regulator of Wnt/*β*-catenin signalling. APC mutations have frequently been found early in the adenoma-to-carcinoma progression of sporadic CRC [28, 29]. Furthermore, APC is mutated in familial adenomatous polyposis (FAP), which facilitates the evolution of CRC [30]. APC binds to various proteins, including *β*-catenin, axin, CtBP, Asefs, IQGAP1, EB1, and microtubules, and is a vital component of the destruction complex in the Wnt/*β*-catenin signalling pathway. Nonsense or frameshift mutations in the *β*-catenin-binding region of APC (MCR: Codons 1267–1529) can shorten the protein, leading to the loss of the capability of APC to bind to *β*-catenin, resulting in activation of cell proliferation and migration [31]. Thus, Wnt/*β*-catenin signalling pathway activation is strongly induced by APC gene mutations, and increases in *β*-catenin expression can promote CRC cell proliferation.

In this study, we identified a different mechanism by which *β*-catenin expression is elevated in CRC cells. Specifically, we confirmed that H19 binds to miR-200a and elevates *β*-catenin expression and activity in CRC cells, a process believed to involve an H19-mediated competing endogenous RNA (ceRNA) pathway. Researchers have recently hypothesized that ceRNAs include mRNAs, lncRNAs, pseudogenes, and other molecules that possess identical miRNA response elements (MREs) that competitively bind to the same miRNA, thereby influencing cell status [32]. According to the ceRNA concept, miRNAs and MREs are two key elements. Specifically, miRNAs bind to the MREs of mRNAs, pseudogenes, and lncRNAs, thereby impacting the posttranscriptional functions of target mRNAs and lncRNAs [33]. Additionally, lncRNAs act as ceRNAs in many types of human cancer. For example, HULC is an lncRNA that is specifically overexpressed in hepatocellular carcinoma (HCC) [34] and functions as a miRNA sponge to restrain the activity of many miRNAs, such as hsa-miR-372-5p, leading to the suppression of its target gene PRKACB. Specifically, HULC acts as the catalytic subunit of PKA, induces the phosphorylation of cAMP response element binding protein (CREB), and promotes CREB-dependent HULC upregulation in HCC [35]. HOTAIR is another lncRNA that plays a role in the development of multiple types of cancer by interacting with Polycomb Repressive Complex 2 (PRC2) [36]. The findings of previous studies indicate that HOTAIR also functions as a ceRNA by promoting human epithelial growth factor receptor 2 (HER2) expression by competitively binding to hsa-miR-331-3p during gastric cancer pathogenesis [37]. Thus, ceRNAs are involved in many types of cancer, such as prostate cancer [38], breast cancer [39, 40], melanoma [41], and glioblastoma [42]. Several recent studies have demonstrated that H19 functions as a ceRNA for miR-138 and miR-200a, antagonizing their functions to cause the derepression of their endogenous targets, namely, Vimentin, ZEB1, and ZEB2, thereby promoting EMT progression in CRC [43]. In this study, we discovered that H19 competitively binds to miR-200a, which removes miR-200a from *β*-catenin and increases *β*-catenin expression.

## 5. Conclusion

H19 is overexpressed in CRC tissues compared with adjacent normal tissues. Furthermore, H19 promotes cell proliferation by competitively binding to miR-200a, thereby upregulating *β*-catenin expression in CRC.

## Figures and Tables

**Table 1:** Primers designed for this study.

Primer name	Primer sequence (5′-3′)
H19 full length	Forward GGATCCAGTTAGAAAAAGCCCGGGCT
	Reverse ACGCGTGCTGTAACAGTGTTTATTGA
WT	Forward CGCGTCTCGTAGTGTTAAGTTATAGA
	Reverse AGCTTCTATAACTTAACACTACGAGA
Mutant	Forward CGCGTCTCGTAGTGTGTAGTTATAGA
	Reverse AGCTTCTATAACTACACACTACGAGA
H19	Forward CTGGGCAACGGAGGTGTA
	Reverse CTGGGAGGGTGTCTGCTTC
miR-200a	Forward TAACACTGTCTGGTAACG
miR-200aRT	GTCGTATCCAGTGCAGGGTCCGAGGTATTCGCACTGGATACGACCGTTACTAATACTGCCTGGTAATGATGA
U6	Forward CTCGCTTCGGCAGCACATATACT
	Reverse ACGCTTCACGAATTTGCGTGTC
a-tubulin	Forward ACCTTAACCGCCTTATTAGCCA
	Reverse ACATTCAGGGCTCCATCAAATC
